# Supplementary figures and images for: A pro-oxidant combination of resveratrol and copper down-regulates hallmarks of cancer and immune checkpoints in patients with advanced oral cancer: Results of an exploratory study (RESCU 004)
Source: Front Oncol. 2022 Sep 16;12:1000957. doi: 10.3389/fonc.2022.1000957 (PMC9525028; doi:10.3389/fonc.2022.1000957)

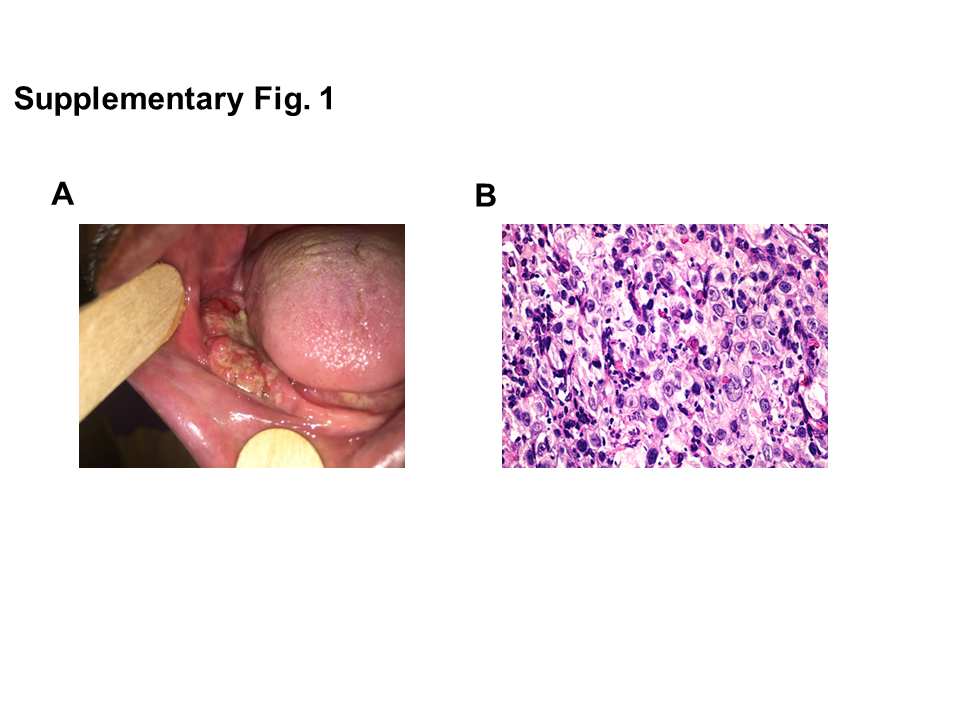

Supplement: Supplementary Figure 1 — A representative photograph of advanced OSCC (A), and its squamous cell phenotype as seen on H&E section (B). [file Image_1.png]

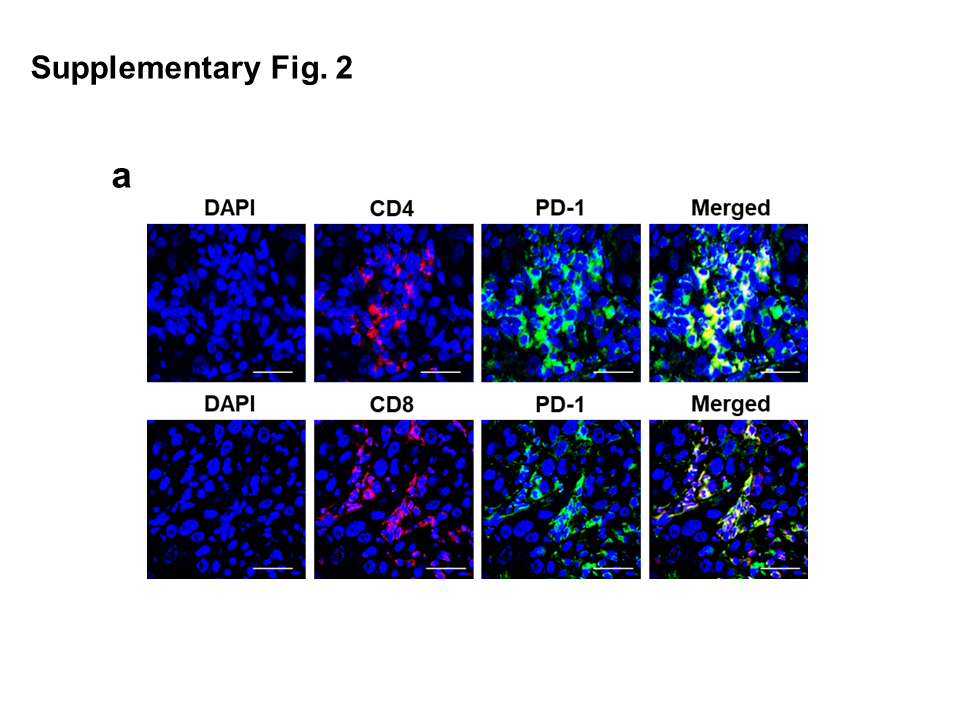

Supplement: Supplementary Figure 2 — Representative images showing expression of various immune checkpoint proteins by CD4 and CD8 lymphocytes. FFPE sections of OSCC tumour tissues were simultaneously immune-stained with antibodies against various immune-checkpoint proteins and those against CD4 and CD8 lymphocytes (scale bar 10µm). Co-localisation of all four immune check-point proteins and CD4 and CD8 lymphocytes is clearly seen. [file Image_2.png]

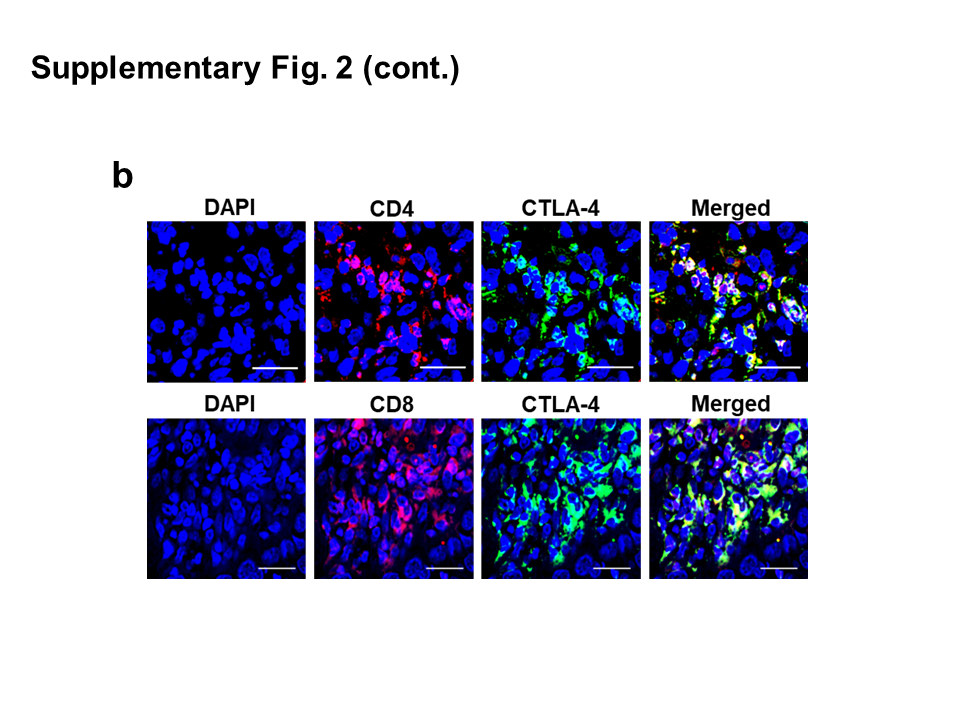

Supplement: Supplementary file 3 [file Image_3.png]

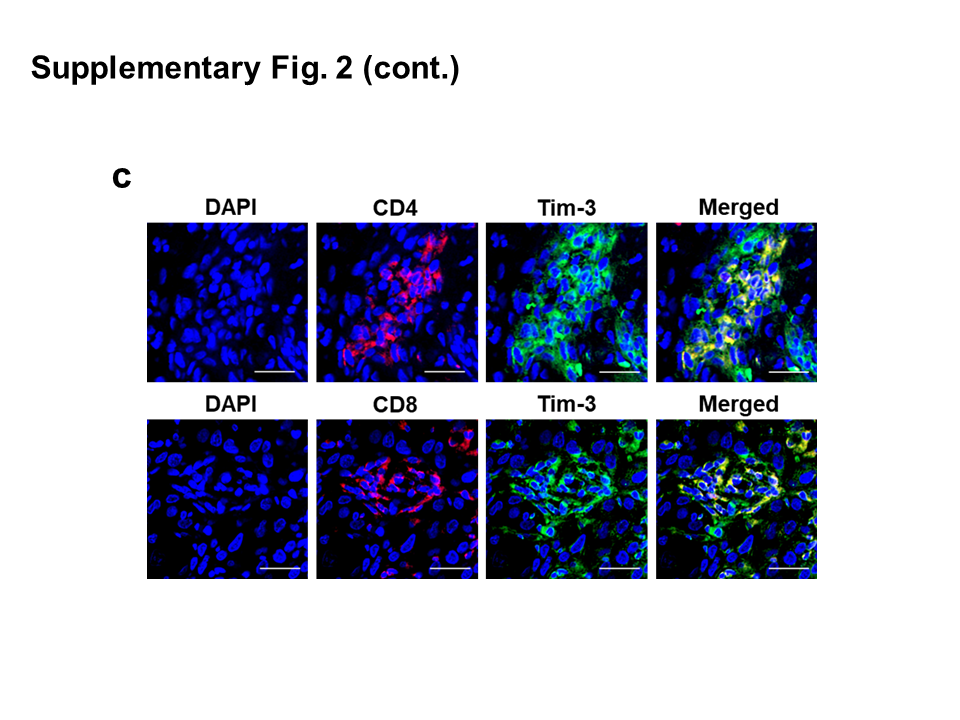

Supplement: Supplementary file 4 [file Image_4.png]

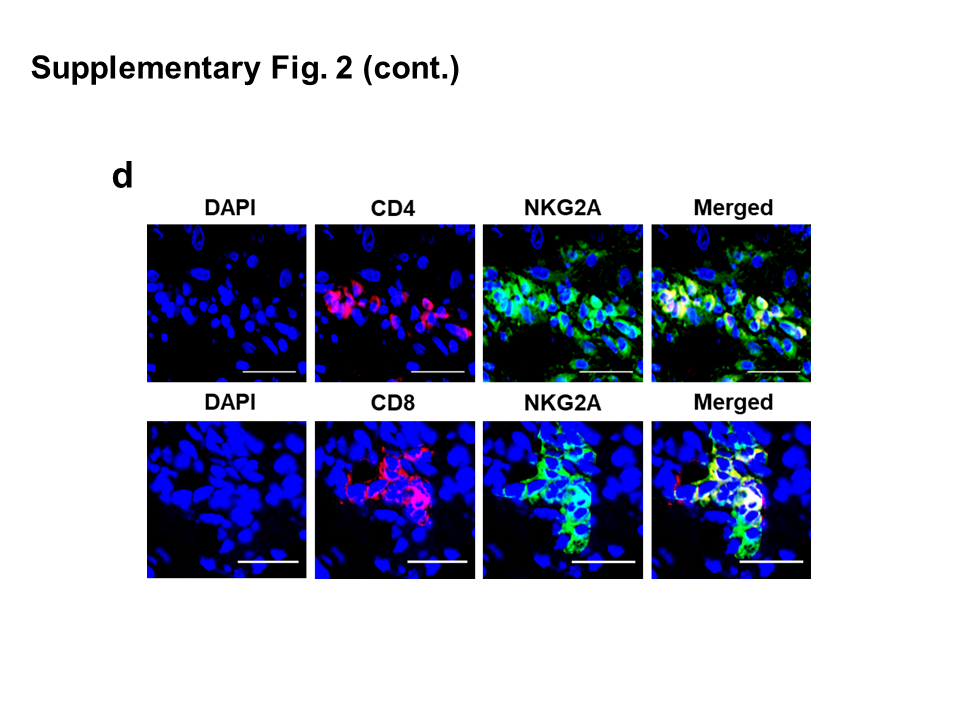

Supplement: Supplementary file 5 [file Image_5.png]
