# Supplementary material for: A pro-oxidant combination of resveratrol and copper down-regulates hallmarks of cancer and immune checkpoints in patients with advanced oral cancer: Results of an exploratory study (RESCU 004)
Source: Front Oncol. 2022 Sep 16;12:1000957. doi: 10.3389/fonc.2022.1000957 (PMC9525028; doi:10.3389/fonc.2022.1000957)
Supplement: Supplementary file 6 [file Table_1.docx]

**Supplementary Table 1:** Demographic and other details.

| **S. No.** | **Dose level** | **Age** | **Sex** | **Site &**  **HP type** | **HP grade** | **TNM stage** |  |
| --- | --- | --- | --- | --- | --- | --- | --- |
| 1 | Control | 40 | MALE | OSCC | MD | T4N1M0 |  |
| 2 | Control | 49 | MALE | OSCC | PD | T4N0M0 |  |
| 3 | Control | 37 | MALE | OSCC | PD | T4N1M0 |  |
| 4 | Control | 42 | MALE | OSCC | MD | T4N3M0 |  |
| 5 | Control | 45 | MALE | OSCC | MD | T4N1M0 |  |
|  | | | | | | | |
| 6 | Dose level 1 | 52 | MALE | OSCC | WD | T4N3M0 |  |
| 7 | Dose level 1 | 65 | MALE | OSCC | MD | T4N0M0 |  |
| 8 | Dose level 1 | 50 | MALE | OSCC | MD | T4N1M0 |  |
| 9 | Dose level 1 | 51 | FEMALE | OSCC | MD | T4N0M0 |  |
| 10 | Dose level 1 | 49 | FEMALE | OSCC | MD | T4N0M0 |  |
|  | | | | | | | |
| 11 | Dose level 2 | 71 | FEMALE | OSCC | MD | T4N0M0 |  |
| 12 | Dose level 2 | 52 | MALE | OSCC | MD | T4N1M0 |  |
| 13 | Dose level 2 | 53 | MALE | OSCC | MD | T4N1M0 |  |
| 14 | Dose level 2 | 69 | MALE | OSCC | MD | T4N1M0 |  |
| 15 | Dose level 2 | 49 | MALE | OSCC | PD | T4N0M0 |  |
|  | | | | | | | |
| 16 | Dose level 3 | 57 | MALE | OSCC | MD | T4N0M0 |  |
| 17 | Dose level 3 | 36 | MALE | OSCC | PD | T4N1M0 |  |
| 18 | Dose level 3 | 46 | MALE | OSCC | PD | T4N1M0 |  |
| 19 | Dose level 3 | 64 | MALE | OSCC | MD | T4N1M0 |  |
| 20 | Dose level 3 | 33 | MALE | OSCC | MD | T4N1M0 |  |
|  | | | | | | | |
| 21 | Dose level 4 | 42 | MALE | OSCC | PD | T4N0M0 |  |
| 22 | Dose level 4 | 36 | MALE | OSCC | MD | T4N1M0 |  |
| 23 | Dose level 4 | 56 | MALE | OSCC | MD | T4N1M0 |  |
| 24 | Dose level 4 | 69 | FEMALE | OSCC | MD | T4N1M0 |  |
| 25 | Dose level 4 | 49 | MALE | OSCC | MD | T4N1M0 |  |

HP type = Histopathological type

HP grade = Histopathological grade

OSCC = Squamous cell carcinoma of oral cavity

WD = Well differentiated; MD = Moderately differentiated; PD = Poorly differentiated
