# Supplementary material for: A pro-oxidant combination of resveratrol and copper down-regulates hallmarks of cancer and immune checkpoints in patients with advanced oral cancer: Results of an exploratory study (RESCU 004)
Source: Front Oncol. 2022 Sep 16;12:1000957. doi: 10.3389/fonc.2022.1000957 (PMC9525028; doi:10.3389/fonc.2022.1000957)
Supplement: Supplementary file 7 [file Table_2.docx]

Supplementary Table 2: Treatment groups and dose

| **Doses Levels** | **Resveratrol** | **Copper** | **Patients (no.)** |
| --- | --- | --- | --- |
| Control | 0 | 0 | 5 |
| Dose level I | 5.6 mg | 560 ng | 5 |
| Dose level II | 50 mg | 5 µg | 5 |
| Dose level III | 500 mg | 50 µg | 5 |
| Dose level IV | 500 mg | 5 mg | 5 |
